# Supplementary figures and images for: Exploring the Significance of Pharmaceutical Care in Mental Health: A Spotlight on Cannabis
Source: Pharmacy (Basel). 2024 Jun 27;12(4):100. doi: 10.3390/pharmacy12040100 (PMC11270281; doi:10.3390/pharmacy12040100)

Figure S1 - PRISMA

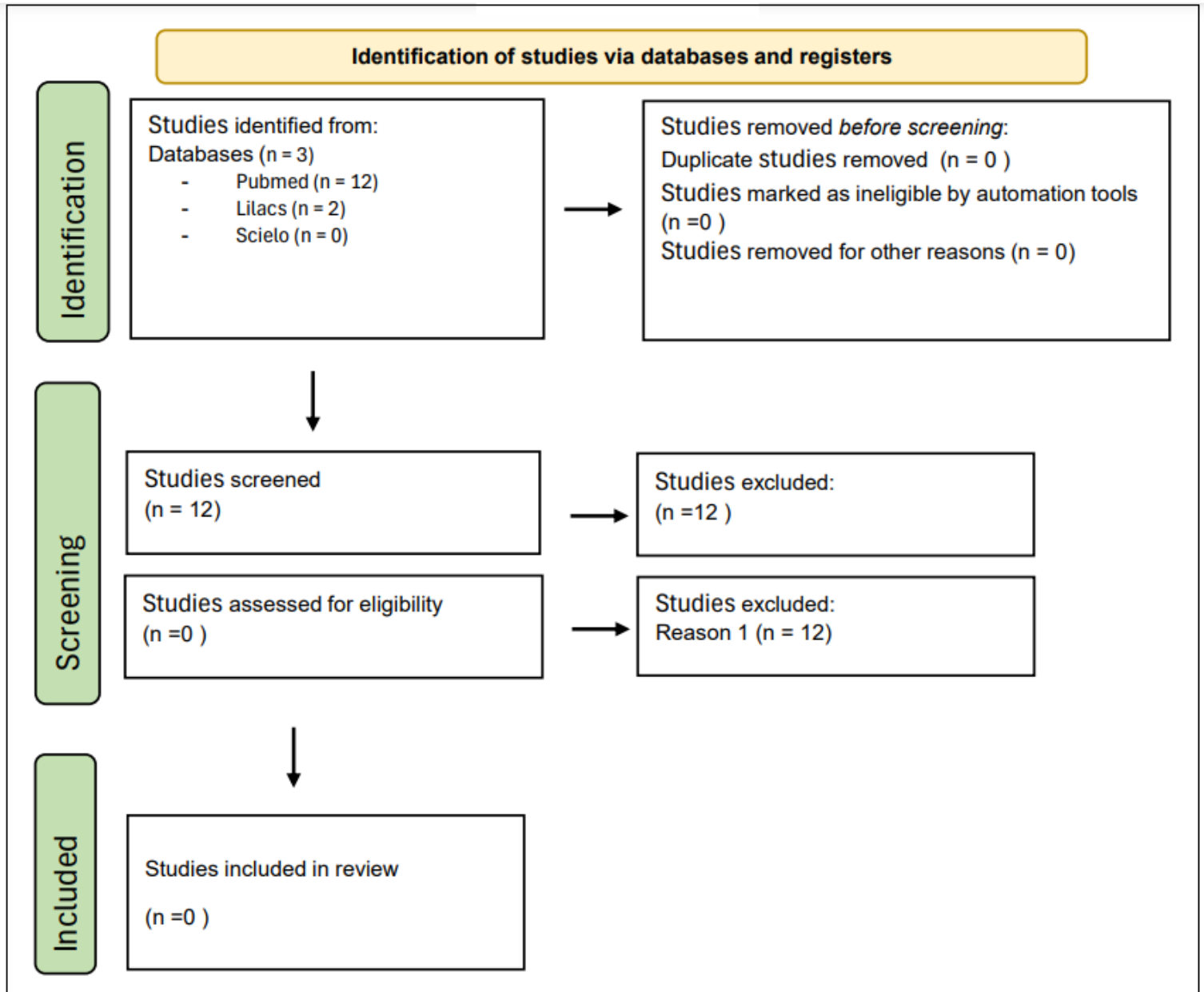

Supplement: Supplementary file 1 [file pharmacy-12-00100-s001.zip › pharmacy-2904267-supplementary.pdf]
